# 2-Methyl-*N*′-(4-nitro­benzyl­idene)benzohydrazide

**DOI:** 10.1107/S1600536810038869

**Published:** 2010-10-02

**Authors:** Chun-Bao Tang

**Affiliations:** a Department of Chemistry, Jiaying University, Meizhou 514015, People’s Republic of China

## Abstract

The title hydrazone compound, C_15_H_13_N_3_O_3_, was prepared by the condensation of 4-nitro­benzaldehyde with 2-methyl­benzohydrazide in methanol. The dihedral angle between the two benzene rings is 14.8 (2)°. In the crystal, mol­ecules are linked through inter­molecular N—H⋯O hydrogen bonds, forming chains along the *a* axis.

## Related literature

For general background to hydrazones, see: Rasras *et al.* (2010 ▶); Pyta *et al.* (2010 ▶); Angelusiu *et al.* (2010 ▶); Fun *et al.* (2008 ▶); Singh & Singh (2010 ▶); Ahmad *et al.* (2010 ▶). For bond-length data, see: Allen *et al.* (1987 ▶). For a similar hydrazone compound reported recently by the author, see: Tang (2010 ▶).
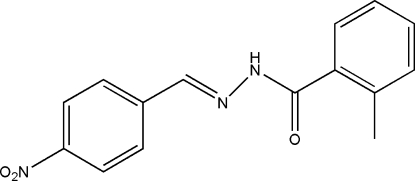


## Experimental

### 

#### Crystal data

C_15_H_13_N_3_O_3_
*M* *_r_* = 283.28
Monoclinic, 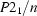

*a* = 7.416 (1) Å
*b* = 26.198 (3) Å
*c* = 7.860 (2) Å
β = 114.206 (1)°
*V* = 1392.8 (4) Å^3^
*Z* = 4
Mo *K*α radiation
μ = 0.10 mm^−1^
*T* = 298 K
0.20 × 0.18 × 0.18 mm

#### Data collection

Bruker SMART CCD area-detector diffractometer
Absorption correction: multi-scan (*SADABS*; Sheldrick, 1996 ▶) *T*_min_ = 0.981, *T*_max_ = 0.983
7336 measured reflections
2941 independent reflections
1696 reflections with *I* > 2σ(*I*)
*R*_int_ = 0.046

#### Refinement

*R*[*F* ^2^ > 2σ(*F* ^2^)] = 0.059
*wR*(*F* ^2^) = 0.140
*S* = 1.01
2941 reflections
194 parameters
1 restraint
H atoms treated by a mixture of independent and constrained refinement
Δρ_max_ = 0.22 e Å^−3^
Δρ_min_ = −0.23 e Å^−3^

### 

Data collection: *SMART* (Bruker, 2002 ▶); cell refinement: *SAINT* (Bruker, 2002 ▶); data reduction: *SAINT*; program(s) used to solve structure: *SHELXS97* (Sheldrick, 2008 ▶); program(s) used to refine structure: *SHELXL97* (Sheldrick, 2008 ▶); molecular graphics: *SHELXTL* (Sheldrick, 2008 ▶); software used to prepare material for publication: *SHELXTL*.

## Supplementary Material

Crystal structure: contains datablocks global, I. DOI: 10.1107/S1600536810038869/rz2493sup1.cif

Structure factors: contains datablocks I. DOI: 10.1107/S1600536810038869/rz2493Isup2.hkl

Additional supplementary materials:  crystallographic information; 3D view; checkCIF report

## Figures and Tables

**Table 1:** Hydrogen-bond geometry (Å, °)

*D*—H⋯*A*	*D*—H	H⋯*A*	*D*⋯*A*	*D*—H⋯*A*
N3—H3⋯O3^i^	0.90 (1)	1.99 (1)	2.870 (2)	167 (3)

Symmetry code: (i) 
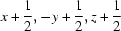
.

**Fig. 1.**
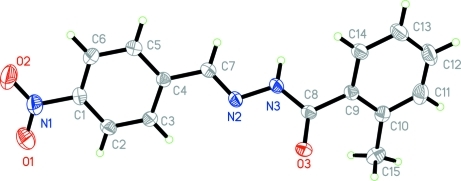
The molecular structure of the title compound, showing the atom-numbering scheme. Displacement ellipsoids are drawn at the 30% probability level.

**Fig. 2.**
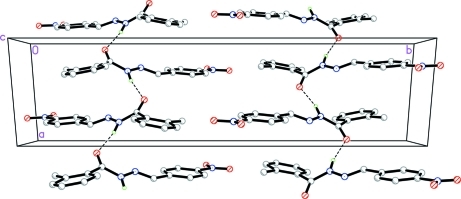
Molecular packing of the title compound viewed along the c axis, with hydrogen bonds shown as dashed lines.

## supplementary crystallographic information

### Comment

Hydrazone compounds have received much attention in biological chemistry and structural chemistry in the last few years (Rasras *et al.*, 2010; Pyta *et al.*, 2010; Angelusiu *et al.*, 2010; Fun *et al.*, 2008; Singh & Singh, 2010; Ahmad *et al.*, 2010). In the present paper, the author reports the crystal structure of the title new hydrazone compound (Fig. 1).

In the title molecule, the dihedral angle between the two benzene rings is 14.8 (2)°. The torsion angles C4—C7—N2—N3, C7—N2—N3—C8 and N2—N3—C8—C9 are 3.9 (2), 13.8 (2), and 1.5 (2)°, respectively. All the bond lengths are within normal values (Allen *et al.*, 1987) and comparable with those of a similar hydrazone compound the author reported recently (Tang, 2010).

In the crystal structure of the compound, molecules are linked through N–H···O intermolecular hydrogen bonds (Table 1), forming chains along the *a* axis (Fig. 2).

### Experimental

4-Nitrobenzaldehyde (0.1 mmol, 15.1 mg) and 3-methylbenzohydrazide (0.1 mmol, 15.0 mg) were dissolved in methanol (20 ml). The mixture was stirred at reflux for 10 min to give a clear yellow solution. Yellow block-shaped crystals of the title compound were formed by slow evaporation of the solvent over several days.

### Refinement

Atom H3 was located in a difference Fourier map and refined isotropically, with the N–H distance restrained to 0.90 (1) Å [*U*_iso_(H) = 0.08 Å^2^]. Other H atoms were constrained to ideal geometries, with C–H = 0.93–0.96 Å, and with *U*_iso_(H) = 1.2*U*_eq_(C) or 1.5*U*_eq_(C) for methyl H atoms.

### Crystal data

**Table:**

C_15_H_13_N_3_O_3_	*F*(000) = 592
*M**_r_* = 283.28	*D*_x_ = 1.351 Mg m^−^^3^
Monoclinic, *P*2_1_/*n*	Mo *K*α radiation, λ = 0.71073 Å
Hall symbol: -P 2yn	Cell parameters from 996 reflections
*a* = 7.416 (1) Å	θ = 2.7–24.5°
*b* = 26.198 (3) Å	µ = 0.10 mm^−^^1^
*c* = 7.860 (2) Å	*T* = 298 K
β = 114.206 (1)°	Block, yellow
*V* = 1392.8 (4) Å^3^	0.20 × 0.18 × 0.18 mm
*Z* = 4	

### Data collection

**Table:**

Bruker SMART CCD area-detector diffractometer	2941 independent reflections
Radiation source: fine-focus sealed tube	1696 reflections with *I* > 2σ(*I*)
graphite	*R*_int_ = 0.046
ω scans	θ_max_ = 27.0°, θ_min_ = 3.0°
Absorption correction: multi-scan (*SADABS*; Sheldrick, 1996)	*h* = −6→9
*T*_min_ = 0.981, *T*_max_ = 0.983	*k* = −28→33
7336 measured reflections	*l* = −10→9

### Refinement

**Table:**

Refinement on *F*^2^	Primary atom site location: structure-invariant direct methods
Least-squares matrix: full	Secondary atom site location: difference Fourier map
*R*[*F*^2^ > 2σ(*F*^2^)] = 0.059	Hydrogen site location: inferred from neighbouring sites
*wR*(*F*^2^) = 0.140	H atoms treated by a mixture of independent and constrained refinement
*S* = 1.01	*w* = 1/[σ^2^(*F*_o_^2^) + (0.0397*P*)^2^ + 0.3174*P*] where *P* = (*F*_o_^2^ + 2*F*_c_^2^)/3
2941 reflections	(Δ/σ)_max_ < 0.001
194 parameters	Δρ_max_ = 0.22 e Å^−^^3^
1 restraint	Δρ_min_ = −0.23 e Å^−^^3^

### Special details

**Table:**

Geometry. All e.s.d.'s (except the e.s.d. in the dihedral angle between two l.s. planes) are estimated using the full covariance matrix. The cell e.s.d.'s are taken into account individually in the estimation of e.s.d.'s in distances, angles and torsion angles; correlations between e.s.d.'s in cell parameters are only used when they are defined by crystal symmetry. An approximate (isotropic) treatment of cell e.s.d.'s is used for estimating e.s.d.'s involving l.s. planes.
Refinement. Refinement of *F*^2^ against ALL reflections. The weighted *R*-factor *wR* and goodness of fit *S* are based on *F*^2^, conventional *R*-factors *R* are based on *F*, with *F* set to zero for negative *F*^2^. The threshold expression of *F*^2^ > σ(*F*^2^) is used only for calculating *R*-factors(gt) *etc*. and is not relevant to the choice of reflections for refinement. *R*-factors based on *F*^2^ are statistically about twice as large as those based on *F*, and *R*- factors based on ALL data will be even larger.

### Fractional atomic coordinates and isotropic or equivalent isotropic displacement parameters (Å^2^)

**Table:**

	*x*	*y*	*z*	*U*_iso_*/*U*_eq_	
N1	0.2497 (4)	0.47404 (10)	−0.4463 (3)	0.0669 (7)	
N2	0.2408 (3)	0.28740 (7)	0.0842 (2)	0.0433 (5)	
N3	0.2780 (3)	0.25697 (8)	0.2388 (3)	0.0448 (5)	
O1	0.2312 (4)	0.45771 (9)	−0.5969 (3)	0.0985 (8)	
O2	0.2575 (5)	0.51917 (9)	−0.4093 (3)	0.1195 (10)	
O3	0.0631 (2)	0.19571 (6)	0.0688 (2)	0.0510 (5)	
C1	0.2623 (3)	0.43743 (9)	−0.3004 (3)	0.0456 (6)	
C2	0.2438 (3)	0.38615 (9)	−0.3415 (3)	0.0470 (6)	
H2	0.2237	0.3747	−0.4599	0.056*	
C3	0.2557 (3)	0.35214 (9)	−0.2037 (3)	0.0440 (6)	
H3A	0.2420	0.3174	−0.2301	0.053*	
C4	0.2877 (3)	0.36894 (8)	−0.0257 (3)	0.0380 (5)	
C5	0.3081 (4)	0.42089 (9)	0.0113 (3)	0.0504 (6)	
H5	0.3316	0.4326	0.1302	0.060*	
C6	0.2938 (4)	0.45538 (9)	−0.1266 (3)	0.0527 (7)	
H6	0.3055	0.4902	−0.1019	0.063*	
C7	0.3044 (3)	0.33305 (9)	0.1220 (3)	0.0431 (6)	
H7	0.3625	0.3436	0.2458	0.052*	
C8	0.1838 (3)	0.21176 (9)	0.2194 (3)	0.0383 (5)	
C9	0.2356 (3)	0.18386 (9)	0.3991 (3)	0.0382 (5)	
C10	0.2726 (3)	0.13129 (9)	0.4118 (3)	0.0440 (6)	
C11	0.3170 (4)	0.10849 (11)	0.5841 (4)	0.0620 (8)	
H11	0.3466	0.0738	0.5981	0.074*	
C12	0.3184 (4)	0.13555 (13)	0.7343 (4)	0.0689 (9)	
H12	0.3460	0.1188	0.8466	0.083*	
C13	0.2796 (4)	0.18702 (12)	0.7209 (3)	0.0620 (8)	
H13	0.2801	0.2052	0.8226	0.074*	
C14	0.2399 (4)	0.21091 (10)	0.5539 (3)	0.0489 (6)	
H14	0.2155	0.2458	0.5437	0.059*	
C15	0.2671 (4)	0.09958 (10)	0.2502 (4)	0.0589 (7)	
H15A	0.1333	0.0970	0.1585	0.088*	
H15B	0.3172	0.0660	0.2935	0.088*	
H15C	0.3474	0.1154	0.1954	0.088*	
H3	0.373 (3)	0.2669 (10)	0.349 (2)	0.080*	

### Atomic displacement parameters (Å^2^)

**Table:**

	*U*^11^	*U*^22^	*U*^33^	*U*^12^	*U*^13^	*U*^23^
N1	0.0865 (18)	0.0589 (17)	0.0603 (16)	0.0004 (13)	0.0353 (14)	0.0137 (13)
N2	0.0457 (12)	0.0447 (12)	0.0331 (10)	−0.0031 (9)	0.0098 (9)	0.0067 (9)
N3	0.0462 (13)	0.0476 (12)	0.0294 (10)	−0.0062 (10)	0.0041 (9)	0.0065 (9)
O1	0.158 (2)	0.0905 (17)	0.0585 (14)	−0.0072 (15)	0.0554 (15)	0.0135 (12)
O2	0.222 (3)	0.0534 (15)	0.1011 (19)	0.0010 (17)	0.084 (2)	0.0192 (14)
O3	0.0561 (11)	0.0503 (10)	0.0322 (9)	−0.0084 (8)	0.0035 (8)	−0.0006 (8)
C1	0.0468 (15)	0.0457 (15)	0.0461 (14)	0.0031 (12)	0.0209 (12)	0.0088 (12)
C2	0.0510 (16)	0.0525 (16)	0.0385 (13)	0.0020 (12)	0.0193 (12)	−0.0007 (12)
C3	0.0493 (15)	0.0384 (13)	0.0427 (14)	0.0021 (11)	0.0172 (12)	−0.0015 (11)
C4	0.0350 (13)	0.0400 (14)	0.0365 (13)	0.0002 (10)	0.0121 (11)	0.0020 (10)
C5	0.0614 (17)	0.0467 (16)	0.0421 (14)	−0.0016 (12)	0.0204 (13)	−0.0029 (12)
C6	0.0657 (18)	0.0376 (14)	0.0555 (16)	−0.0021 (12)	0.0255 (14)	−0.0009 (12)
C7	0.0437 (14)	0.0463 (15)	0.0343 (13)	−0.0003 (11)	0.0109 (11)	0.0007 (11)
C8	0.0382 (13)	0.0418 (14)	0.0309 (12)	0.0019 (11)	0.0101 (11)	−0.0012 (10)
C9	0.0310 (12)	0.0467 (15)	0.0322 (12)	−0.0024 (10)	0.0081 (10)	0.0031 (10)
C10	0.0331 (13)	0.0458 (15)	0.0479 (14)	−0.0018 (11)	0.0113 (11)	0.0043 (11)
C11	0.0518 (17)	0.0580 (17)	0.0681 (19)	−0.0012 (14)	0.0162 (15)	0.0195 (15)
C12	0.0616 (19)	0.090 (2)	0.0449 (16)	−0.0146 (17)	0.0116 (14)	0.0229 (16)
C13	0.0640 (19)	0.085 (2)	0.0356 (14)	−0.0184 (16)	0.0189 (13)	−0.0034 (14)
C14	0.0499 (15)	0.0561 (16)	0.0381 (13)	−0.0060 (12)	0.0156 (12)	−0.0016 (12)
C15	0.0514 (17)	0.0499 (16)	0.0742 (19)	0.0054 (13)	0.0246 (15)	−0.0065 (14)

### Geometric parameters (Å, °)

**Table:**

N1—O1	1.213 (3)	C6—H6	0.9300
N1—O2	1.213 (3)	C7—H7	0.9300
N1—C1	1.468 (3)	C8—C9	1.494 (3)
N2—C7	1.275 (3)	C9—C14	1.397 (3)
N2—N3	1.383 (2)	C9—C10	1.400 (3)
N3—C8	1.351 (3)	C10—C11	1.391 (3)
N3—H3	0.900 (10)	C10—C15	1.504 (3)
O3—C8	1.229 (2)	C11—C12	1.373 (4)
C1—C6	1.371 (3)	C11—H11	0.9300
C1—C2	1.375 (3)	C12—C13	1.374 (4)
C2—C3	1.377 (3)	C12—H12	0.9300
C2—H2	0.9300	C13—C14	1.373 (3)
C3—C4	1.391 (3)	C13—H13	0.9300
C3—H3A	0.9300	C14—H14	0.9300
C4—C5	1.387 (3)	C15—H15A	0.9600
C4—C7	1.459 (3)	C15—H15B	0.9600
C5—C6	1.382 (3)	C15—H15C	0.9600
C5—H5	0.9300		
			
O1—N1—O2	123.6 (3)	O3—C8—N3	123.2 (2)
O1—N1—C1	118.5 (3)	O3—C8—C9	123.1 (2)
O2—N1—C1	117.9 (2)	N3—C8—C9	113.66 (19)
C7—N2—N3	114.48 (19)	C14—C9—C10	120.2 (2)
C8—N3—N2	119.86 (18)	C14—C9—C8	118.7 (2)
C8—N3—H3	121.8 (18)	C10—C9—C8	121.0 (2)
N2—N3—H3	118.2 (18)	C11—C10—C9	116.9 (2)
C6—C1—C2	121.9 (2)	C11—C10—C15	119.9 (2)
C6—C1—N1	119.0 (2)	C9—C10—C15	123.1 (2)
C2—C1—N1	119.1 (2)	C12—C11—C10	122.1 (3)
C1—C2—C3	118.6 (2)	C12—C11—H11	119.0
C1—C2—H2	120.7	C10—C11—H11	119.0
C3—C2—H2	120.7	C11—C12—C13	120.9 (3)
C2—C3—C4	121.0 (2)	C11—C12—H12	119.5
C2—C3—H3A	119.5	C13—C12—H12	119.5
C4—C3—H3A	119.5	C14—C13—C12	118.5 (3)
C5—C4—C3	118.7 (2)	C14—C13—H13	120.8
C5—C4—C7	119.9 (2)	C12—C13—H13	120.8
C3—C4—C7	121.3 (2)	C13—C14—C9	121.4 (2)
C6—C5—C4	120.7 (2)	C13—C14—H14	119.3
C6—C5—H5	119.7	C9—C14—H14	119.3
C4—C5—H5	119.7	C10—C15—H15A	109.5
C1—C6—C5	119.0 (2)	C10—C15—H15B	109.5
C1—C6—H6	120.5	H15A—C15—H15B	109.5
C5—C6—H6	120.5	C10—C15—H15C	109.5
N2—C7—C4	121.1 (2)	H15A—C15—H15C	109.5
N2—C7—H7	119.5	H15B—C15—H15C	109.5
C4—C7—H7	119.5		

### Hydrogen-bond geometry (Å, °)

**Table:**

*D*—H···*A*	*D*—H	H···*A*	*D*···*A*	*D*—H···*A*
N3—H3···O3^i^	0.90 (1)	1.99 (1)	2.870 (2)	167 (3)

Symmetry codes: (i) *x*+1/2, −*y*+1/2, *z*+1/2.
